# Rapidly Fatal Puerperal Peritonitis—Atmospheric Constitution of 1825-6-7-8

**Published:** 1829-01-01

**Authors:** 


### XXVII.

### RAPIDLY FATAL PUERPERAL PERITONITIS. ATMOSPHERIC CONSTITUTION.

A case of the above kind is communicated by Mr. Dalrymple, of Norwich, to Dr. Farre. We shall state the particulars, before offering any remarks.

A lady, in the 30th year of her age, was delivered of a fine boy, (the fourth child) on the morning of Dec. 17. The three preceding labours had been painful and protracted, the last of the three requiring instrumental aid. On the present occasion, the child was delivered before the arrival of the surgeon. The labour had been rapid and severe. The surgeon left his patient in a tranquil state. At 4, P.M. he again visited her, and all was quiet. An anodyne was prescribed for the night, but was not taken, as the night was spent in easy sleep. A dose of castor oil was taken the next morning, and had acted mildly. At 1, A.M. of the 19th, (the third day, medically computed) the surgeon was summoned in haste. He learnt that, at 8 o'clock in the preceding evening, the patient had been seized with a *rigor* and violent pain. He found her with a burning skin—pulse 130, harsh and full—excruciating pain in the belly, which was tense, tumid, and tender to the touch—uterus risen out of the pelvis, and forming a defined tumour in the hypogastrium. Venesectio ad ℥xviij.—mercurial and saline purge—some relief. In six hours more, the surgeon was again summoned. The skin was dry and parched—pulse quickened, but reduced in force—abdomen still more tumid, and incapable of bearing the slightest pressure. No urine having been passed since the preceding evening, a catheter was introduced, but no water was found in the bladder. Twenty-four leeches above the pubes—warm fomentations—blister to the abdomen—50 drops of Battley's liquor opii sedativus. *Evening.* Much the same state—pain of abdomen rather less urgent, but its sensibility to pressure the same. Patient lies on her back, with her legs extended. Mercurials, aperients, hyosciamus. *20th. Morning.* Evidently sinking—died at 5 in the afternoon.

*Dissection.* The uterus was at least one third larger than usual at this period *post partum*—its peritoneal surface of a light pink colour—the parietes of the uterus very thick, firm, and rather pale—no organic lesion of this viscus. There were slight traces of inflammation observable along the peritoneal surface of the fallopian tubes, as far as the ovaries, which were larger and softer than natural. No trace of inflammation on the peritoneum, or in any other organ or part of the abdomen, with the exception of the right kidney, "which afforded proofs of vascular congestion."

"Here," says Mr. Dalrymple, "is a case entire and complete in all its parts, commencing instantaneously, and with great violence, at a moment when the general system seemed to be in a state of perfect ease and repose; proceeding with frightful rapidity, and terminating speedily, and almost without check, in death.

"Through the great good sense of the friends of the deceased, permission was obtained to inspect the state of the parts; an opportunity most rare and precious in such cases. Suffice it then to observe, that within the space of six or eight weeks, no less than seven cases of fatal puerperal inflammation have either fallen under the immediate personal observation of the reporter of this case, or have been communicated to him; all terminating unfavourably within the short period of fifty hours. Can season, or atmospheric causes, or the 'constitution of the year,' account for this remarkable fatality? In reference to this point, it may not be impertinent to state, on the authority of the intelligent husband of the subject of our case, that an unusual disease has recently prevailed in the dairy department of a large farm in his own occupation; a certain number of cows, calving within a short period, being attacked, the greater part of them, with a malady resembling, so far as we have been capable of judging, the puerperal inflammation, or fever of the human female. Under this disease, the suffering animals were uniformly treated by full and repeated blood-lettings, and all of them eventually recovered.

"A singular coincidence is worthy of remark in this case. The unfortunate subject of this paper took a considerable interest in the treatment and cure of these animals; possibly associating in her own mind the analogy of their disorder with the sufferings which she had experienced in former labours, and which her then similar condition might lead her to anticipate.

"May it not now be asked, whether moral causes may not have so modified the influence and agency of physical causes as to have produced that depressing effect on the general system, which the negative character of the appearances after death has so totally failed to explain?"

We doubt whether the term *peritonitis* be strictly applicable to the above case. Was it a "serious affection occurring after delivery," as described by Dr. Marshall Hall? Was it a puerperal fever modified by the malarious constitution of the years 1826, 1827, and 1828? The following comments of Dr. Farre are highly deserving of attention, for the atmospheric constitution to which he alludes is in full force at this moment.

"If Sydenham had flourished in this era he would have termed the constitution of the years 1825, 6, 7, and 8, Intermittent, and it has been combined with an unusual degree of neuralgic affection, from Hemicrania to the most intractable forms of Tic douloureux. A similar combination of disease took place in the year 1809, but has not prevailed for a quarter of a century to the extent which it has done during the last two years. It commenced in the spring of 1825, having been preceded by an unusually humid autumn, during which the north of Europe was flooded to a very alarming extent. It was ushered in by a very fatal peritonitis, both simple and puerperal, in the human subject, and by a still more destructive wet rot amongst sheep."

"It was at the end of February in that year (1825) that an experienced surgeon at the west end of London said to him, that he had lost four patients in the puerperal state during that month, of what appeared to him to be peritonitis previous to the post-mortem examination, which did not explain to his satisfaction the nature of the disease, he having found, in two of the cases, only a little bloody serum extravasated into the peritoneum. He added, that he could not understand it, and was persuaded that more would be heard of it. His prediction was verified. At the east end of London, not far from the river, this disease proved still more fatal during the month of March. One surgeon informed the Editor that he had lost seven, another four, in all of which the disease was treated at the instant of its formation by active bloodletting. A physician-accoucheur, who attended in consultation many of these cases, stated to him, that out of thirteen cases eleven died, that all which had been bled died, and that the only two which recovered had not been bled, having been treated by turpentine. The summer proved remarkably dry and hot: during July not a drop of rain fell in London. The intermittents commenced with anomalous inflammatory affections, lapsing into continued fever, and sliding into a quotidian or tertian type, rheumatism, subject to dangerous metastasis, erysipelas of an untoward kind, and some cases of malignant sore throat, which had not occurred for a long period before. The autumnal season was humid, and the continued fevers, falsely called typhoid, and approximating more to the remittent form, proved insidious, the fatal collapse occurring early and unexpectedly. The following summer proved still more dry, there being scarcely any rain even in June, and not less hot, so that the pulse crop failed. The character of disease was less malignant, but the same type was still preserved. Puerperal peritonitis was much less fatal, but even in this year one surgeon at the east end of the town lost seven women, in a very few weeks, under a varied treatment. 1827 being rather cold and humid, intermittents, remittents, and neuralgic affections prevailed in their greatest extent; and to conclude in the language of Sydenham, the constitution of the year has not changed in 1828. This fatal peritoneal disease appears to have borne the same relation to peritonitis that the pneumonia notha does to pneumonia, not only in the serous, or sero-sanguineous effusion, instead of coagulable lymph, but also in the complete inability of the patient to bear the lancet."*

We recommend the foregoing observations to the attentive consideration of practitioners. They entirely accord with our own experience—and they may tend to check the effusion of blood during a constitution of the atmosphere which engenders a host of diseases, aping the purely inflammatory, but bearing very badly the vigorous depletion to which we have been accustomed for many years past.

---

### XXVIII.

### ANEURISM OF THE MIDDLE MENINGEAL ARTERY—FATAL OPERATION. By DR. KRIMER.

* Journal of Morbid Anatomy.

